# CKD stage-specific utility of two equations for predicting 1-year risk of ESKD

**DOI:** 10.1371/journal.pone.0293293

**Published:** 2023-11-01

**Authors:** Sijie Zheng, Rishi V. Parikh, Thida C. Tan, Leonid Pravoverov, Jignesh K. Patel, Kate M. Horiuchi, Alan S. Go

**Affiliations:** 1 Department of Nephrology, Kaiser Permanente Oakland Medical Center, Oakland, California, United States of America; 2 Division of Research, Kaiser Permanente Northern California, Oakland, California, United States of America; 3 Department of Medicine, University of California, San Francisco, San Francisco, California, United States of America; 4 Department of Nephrology, Kaiser Permanente Sacramento Medical Center, Sacramento, California, United States of America; 5 Department of Health Systems Science, Kaiser Permanente Bernard J. Tyson School of Medicine, Pasadena, California, United States of America; 6 Departments of Epidemiology, Biostatistics and Medicine, University of California, San Francisco, San Francisco, California, United States of America; 7 Department of Medicine, Stanford University School of Medicine, Stanford, California, United States of America; Cleveland Clinic, UNITED STATES

## Abstract

**Background:**

The Kidney Failure Risk Equation (KFRE) and Kaiser Permanente Northwest (KPNW) models have been proposed to predict progression to ESKD among adults with CKD within 2 and 5 years. We evaluated the utility of these equations to predict the 1-year risk of ESKD in a contemporary, ethnically diverse CKD population.

**Methods:**

We conducted a retrospective cohort study of adult members of Kaiser Permanente Northern California (KPNC) with CKD Stages 3–5 from January 2008-September 2015. We ascertained the onset of ESKD through September 2016, and calculated stage-specific estimates of model discrimination and calibration for the KFRE and KPNW equations.

**Results:**

We identified 108,091 eligible adults with CKD (98,757 CKD Stage 3; 8,384 CKD Stage 4; and 950 CKD Stage 5 not yet receiving kidney replacement therapy), with mean age of 75 years, 55% women, and 37% being non-white. The overall 1-year risk of ESKD was 0.8% (95%CI: 0.8–0.9%). The KFRE displayed only moderate discrimination for CKD 3 and 5 (c = 0.76) but excellent discrimination for CKD 4 (c = 0.86), with good calibration for CKD 3–4 patients but suboptimal calibration for CKD 5. Calibration by CKD stage was similar to KFRE for the KPNW equation but displayed worse calibration across CKD stages for 1-year ESKD prediction.

**Conclusions:**

In a large, ethnically diverse, community-based CKD 3–5 population, both the KFRE and KPNW equation were suboptimal in accurately predicting the 1-year risk of ESKD within CKD stage 3 and 5, but more accurate for stage 4. Our findings suggest these equations can be used in1-year prediction for CKD 4 patients, but also highlight the need for more personalized, stage-specific equations that predicted various short- and long-term adverse outcomes to better inform overall decision-making.

## Introduction

An estimated 15% of Americans are estimated to have CKD, in 2019, 134,608 individuals were newly diagnosed with end-stage kidney disease (ESKD) [1, 2]. For many patients initiating chronic dialysis, the process is not optimally coordinated, and a significant proportion of patients feel under-prepared for dialysis. Unfortunately, the transition period immediately after dialysis initiation has a very high risk of death and hospitalization [1, 3]. Improving the transition from CKD to ESKD represents an important opportunity to improve outcomes and potentially lower healthcare costs. There is also greater appreciation that decline in eGFR is often not linear, with many patients staying in an earlier CKD stage (i.e., stage 3) much longer than in later stages (i.e., stage 4–5) [4], and some progressing rapidly due to occurrence of an episode of acute kidney injury (AKI) [5, 6]. In CKD Stages 4–5, a linear decline in eGFR may occur in <40% of patients [5]. Therefore, accurate ESKD prediction is crucial to delineate patients with a high short-term risk of progressing to ESKD rapidly.

Two efforts to improve ESKD risk prediction include the Kidney Failure Risk Equation (KFRE) and the Kaiser Permanente Northwest (KPNW) equation, which focus only on 2- and 5-year estimates for combined CKD Stages 3–5 [7, 8]. However, both equations have limitations that affect their utility in contemporary practice. For example, the development and validation efforts have been performed in older, primarily white/European individuals, so it remains unclear how generalizable the results are to the increasingly diverse populations in the U.S. KFRE uses four variables (age, sex, eGFR and urine albumin-to-creatinine ratio) [7] and the KPNW equation uses eight variables (age, sex, eGFR, proteinuria/albuminuria, systolic blood pressure, use of antihypertensive medications, hemoglobin level and a diabetes severity index) [8], which may not include all relevant risk factors to accurately estimate the absolute risk of ESKD for individual patients [9]. Given the models’ 2- or 5-year timeframe, they may be mostly applicable in the primary care setting, but potentially less useful for nephrologists when personalizing planning for patients most likely to progress to ESKD within a shorter time frame. The Kidney Disease: Improving Global Outcomes (KDIGO) guidelines recommend timely nephrology referral for planning renal replacement therapy (RRT) in persons whose risk of kidney failure within 1 year is 10–20% or higher [10]. The 2019 update of KDOQI Clinical Practice Guideline for Vascular Access emphasizes a patient-centered care approach that promotes optimal dialysis access management based on the best available evidence and patient’s life expectance and preference. The "ESKD Life-Plan" is achieved by creating a "P-L-A-N" (Patient Life-Plan First then Access Needs) for each patient that considers the patient’s life plan and corresponding access needs [11]. This highlights the importance of having validated and well-calibrated 1-year ESKD risk prediction tools [12].

To address this key knowledge gap, we evaluated the performance of ESKD prediction models for predicting 1-year risk of ESKD by CKD stage within a large, ethnically diverse community-based population.

## Methods

### Source population

The source population was based within Kaiser Permanente Northern California (KPNC), a large, integrated health care delivery system currently caring for >4.6 million members in Northern and Central California. Its membership is highly diverse and representative of the local surrounding and statewide population across demographic characteristics [13]. Nearly all aspects of care are also captured through KPNC’s comprehensive electronic health records (EHR).

The study was approved by the Kaiser Permanente Northern California institutional review board. A waiver of informed consent was obtained due to the retrospective, data-only nature of the study.

### Study sample

We identified all members who were ≥18 years old with at least 2 measurements of outpatient, non-emergency department estimated glomerular filtration rate (eGFR) <60 mL/min/1.73m^2^ calculated using the original serum creatinine-based CKD-EPI equation [14] between January 2008 and September 2015. We defined the index date as the earliest qualifying eGFR measurement during the study period after which all subsequent measurements were <60 mL/min/1.73m^2^ to ensure the patient had CKD. We also required patients to have a qualifying eGFR measurement within 90–365 days after their index date. We excluded patients who received chronic RRT before their index date using data from a comprehensive health system ESKD Registry (i.e., chronic hemodialysis, chronic peritoneal dialysis, or receipt of renal transplant) [15–17]. Lastly, we excluded members with <12 months of continuous health plan membership and pharmacy benefit before their index date to ensure more complete capture of relevant comorbidities, prescriptions, and baseline laboratory measurements.

To assess the validity of the KFRE and the KPNW equation in adult KPNC members with CKD, we applied additional exclusion criteria specific to each equation. For KFRE, we excluded patients with no baseline proteinuria measurements (urine albumin-to-creatinine ratio [uACR], urine protein-to-creatinine ratio [uPCR], or urine dipstick). For the KPNW equation, we additionally excluded patients without baseline outpatient hemoglobin or systolic blood pressure values.

### Follow-up and outcomes

The primary outcome was occurrence of ESKD during up to 1-year follow-up, with censoring at the end of follow-up or earlier if they disenrolled from the health plan or died, based on comprehensive health plan administrative databases, proxy reporting, Social Security Administration vital status files and California state death certificate files [18, 19]. Secondary outcomes included all-cause death based on the data resources described, as well as receipt of hospice services ascertained from the EHR.

### Covariates

Relevant covariates were obtained from EHR-based data that has been standardized and linked at the patient level into the KPNC Virtual Data Warehouse (VDW) [20–25]. Outpatient eGFR was obtained within one year before index date, and urine protein measures up to four years before index date. For patients with missing uACR results, we imputed uACR from available uPCR and urine dipstick values, if available [26]. Demographic characteristics and tobacco use were ascertained from health plan databases [27, 28]. Prior AKI was defined using serum creatinine-based KDIGO criteria [29] within hospitalizations in the 5 years before index date. Relevant comorbidities and cardiac procedures were defined based on inpatient and ambulatory diagnoses and procedures using *International Classification of Disease*, *Ninth and Tenth Edition* (ICD-9 and ICD-10) codes (codes available upon request), relevant laboratory test results, and pharmacy dispensings within 5 years before index date [20–25]. We calculated a Diabetes Complications Severity Index based on diagnostic ICD-9 codes as required for the KPNW equation [8, 30]. We ascertained diabetes mellitus based on diagnoses or procedures using ICD-9 and ICD-10 codes, laboratory results, and filled outpatient prescriptions from hospitalization, ambulatory visit, laboratory, and pharmacy databases. We obtained baseline vital signs from the most recent outpatient clinic visit within one year before index date. For patients with missing baseline hemoglobin and vital signs, we created separate categories for unknown values.

### Statistical approach

All analyses were conducted using SAS, version 9.4 (Cary, N.C.). We compared baseline characteristics across outcome categories. Given the large sample size, we compared standardized mean and proportion differences between groups using Cohen’s D for continuous and binary variables and Cramer’s V for categorical variables. Differences were considered potentially clinically significant if the effect size estimate was ≥0.10. We calculated crude risks and associated 95% confidence intervals for ESKD, use of hospice services, and all-cause death, overall and stratified by CKD stage.

We used the four-variable KFRE (age, eGFR, sex, and uACR) to calculate the 1-year predicted risk of ESKD [7]. For the KPNW equation, we calculated a risk score using age, sex, eGFR, uACR, systolic blood pressure, anti-hypertensive medication usage, hemoglobin, and diabetes complications severity index as categorical variables. KPNW 2-year predicted risk of ESKD was generated from these risk scores using published thresholds [8] and a point estimate conversion table (E. Schroeder, personal communication). To generate a KPNW 1-year risk estimate, we assumed a constant hazard and divided the 2-year risk estimates in half.

Equation discrimination was evaluating using c statistics, and calibration assessed through plots of observed vs. predicted 1-year risk of ESKD within each decile of predicted risk as well as the Brier score [9].

## Results

### Study cohort

**Fig 1:**
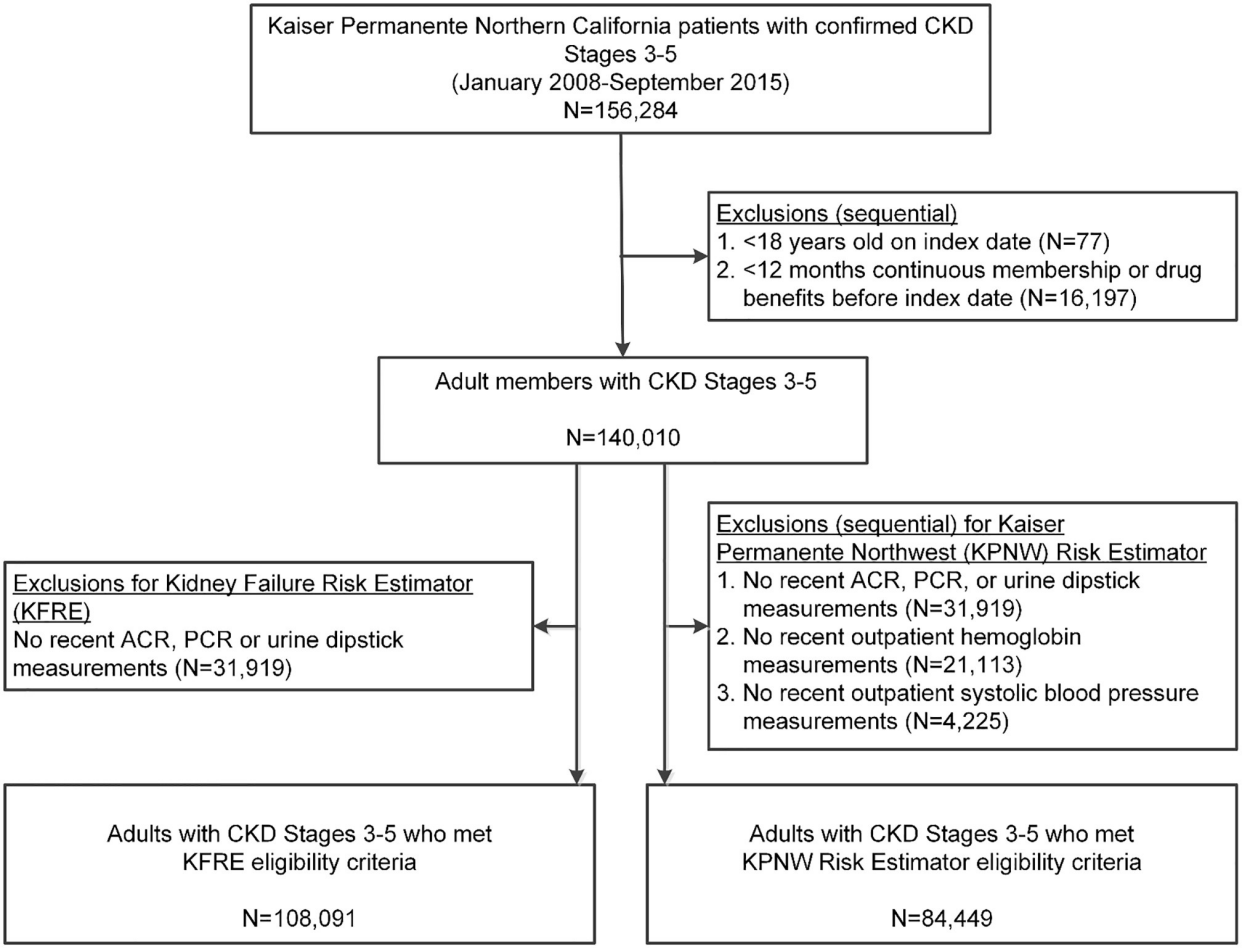
Assembly of adults with CKD stages 3–5 eligible for evaluation using the KFRE and KPNW ESKD risk equations.

We identified 140,010 eligible CKD patients, with 108,091 (91.4% CKD 3, 7.8% CKD 4 and 0.9% CKD 5) eligible for KFRE and 84,449 (90.6% CKD 3, 8.4% CKD 4 and 1.0% CKD 5) eligible for the KPNW equation (Fig 1). Among the 108,091 patients eligible for the KFRE, mean age was 75 years, 54.9% were women, 36.8% were of non-white race, and 12.4% were of Hispanic ethnicity.

Patients who progressed to ESKD within 1 year were more likely to be younger; men; of a minority race; Hispanic; current smokers; have a prior history of AKI, acute myocardial infarction, heart failure, cardiac procedures, diabetes, and chronic liver disease; and have a higher baseline systolic blood pressure and lower baseline hemoglobin than patients who did not progress to ESKD (Table 1). In addition, patients who progressed to ESKD were less likely to have known atrial fibrillation or flutter, thyroid disease, chronic lung disease, and diagnosed dementia and depression (Table 1). Characteristics of the cohort eligible for the KPNW equation are shown in S1 Table.

**Table 1:** Baseline characteristics of 108,091 adults with chronic kidney disease eligible for the KFRE, January 1, 2008-September 30, 2015, overall and stratified by initiation of renal replacement therapy (RRT) within 1 year.

Characteristic	Overall (N = 108,091)	ESKD at 1 year (N = 879)	No ESKD at 1 year (N = 107,212)	Standardized difference*
Age, Mean (SD), years	74.5 (11.0)	62.0 (14.4)	74.6 (10.9)	**1.16**
Age Category, years, n (%)				**1.05**
18–40	843 (0.8)	73 (8.3)	770 (0.7)	
41–60	11,228 (10.4)	330 (37.5)	10,898 (10.2)	
61–75	43,274 (40.0)	315 (35.8)	42,959 (40.1)	
>75	52,746 (48.8)	161 (18.3)	52,585 (49.0)	
Women	59,377 (54.9)	397 (45.2)	58,980 (55.0)	**0.20**
Self-reported Race, n (%)				
White	68,309 (63.2)	332 (37.8)	67,977 (63.4)	**0.44**
Black	8279 (7.7)	131 (14.9)	8148 (7.6)	
Asian/Pacific Islander	13,014 (12.0)	185 (21.0)	12,829 (12.0)	
Other	18,141 (16.8)	230 (26.2)	17,911 (16.7)	
Unknown	348 (0.3)	1 (0.1)	347 (0.3)	
Hispanic ethnicity, n (%)	13,372 (12.4)	210 (23.9)	13,162 (12.3)	**0.35**
Smoking status, n (%)				**0.11**
Current smoker	5837 (5.4)	97 (11.0)	5740 (5.4)	
Former smoker	41,558 (38.4)	298 (33.9)	41,260 (38.5)	
Nonsmoker	60,696 (56.2)	484 (55.1)	60,212 (56.2)	
**Medical history, n (%)**				
Prior acute kidney injury	16,301 (15.1)	276 (31.4)	16,025 (14.9)	**0.58**
Acute myocardial infarction	4170 (3.9)	53 (6.0)	4117 (3.8)	**0.29**
Heart failure	15,856 (14.7)	180 (20.5)	15,676 (14.6)	**0.25**
Ischemic stroke or transient ischemic attack	4951 (4.6)	43 (4.9)	4908 (4.6)	0.04
Peripheral artery disease	8646 (8.0)	60 (6.8)	8586 (8.0)	**0.10**
Mitral and/or aortic valvular disease	9265 (8.6)	73 (8.3)	9192 (8.6)	0.02
Atrial fibrillation or flutter	15,043 (13.9)	80 (9.1)	14,963 (14.0)	**0.29**
Venous thromboembolism	1431 (1.3)	11 (1.3)	1420 (1.3)	0.03
Other thromboembolic events	1257 (1.2)	18 (2.0)	1239 (1.2)	**0.35**
Coronary artery bypass surgery	2326 (2.2)	23 (2.6)	2303 (2.1)	**0.12**
Percutaneous coronary intervention	4247 (3.9)	52 (5.9)	4195 (3.9)	**0.26**
Diabetes mellitus	52,924 (49.0)	593 (67.5)	52,331 (48.8)	**0.47**
Diabetes Complications Severity Index				**0.80**
No Diabetes	45,132 (41.8)	56 (6.4)	45,076 (42.0)	
0 complication	20,090 (18.6)	70 (8.0)	20,020 (18.7)	
1 complication	15,126 (14.0)	135 (15.4)	14,991 (14.0)	
2 complications	8893 (8.2)	251 (28.6)	8642 (8.1)	
> 3 complications	4268 (3.9)	339 (38.6)	3929 (3.7)	
Hypertension	94,522 (87.4)	783 (89.1)	93,739 (87.4)	**0.10**
Dyslipidemia	90,364 (83.6)	743 (84.5)	89,621 (83.6)	0.04
Hyperthyroidism	5681 (5.3)	34 (3.9)	5647 (5.3)	**0.20**
Hypothyroidism	22,592 (20.9)	130 (14.8)	22,462 (21.0)	**0.26**
Chronic liver disease	2914 (2.7)	74 (8.4)	2840 (2.6)	**0.74**
Chronic lung disease	26,891 (24.9)	179 (20.4)	26,712 (24.9)	**0.16**
Diagnosed dementia	5528 (5.1)	7 (0.8)	5521 (5.1)	**1.16**
Diagnosed depression	18,144 (16.8)	129 (14.7)	18,015 (16.8)	**0.10**
Hospitalized bleed	4155 (3.8)	45 (5.1)	4110 (3.8)	**0.18**
Body mass index, Mean (SD), kg/m^2^	29.1 (6.6)	30.1 (7.5)	29.1 (6.6)	**0.15**
Body mass index category, kg/m^2^, n (%)				0.07
<18.5	1445 (1.3)	6 (0.7)	1439 (1.3)	
18.5–24.9	26,390 (24.4)	222 (25.3)	26,168 (24.4)	
25–29.9	35,705 (33.0)	245 (27.9)	35,460 (33.1)	
30–39.9	32,656 (30.2)	285 (32.4)	32,371 (30.2)	
≥40	4672 (4.3)	63 (7.2)	4609 (4.3)	
Unknown	7223 (6.7)	58 (6.6)	7165 (6.7)	
Systolic blood pressure, Mean (SD), mmHg	129.7 (17.8)	140.1 (23.3)	129.7 (17.7)	**0.59**
Systolic blood pressure category, mmHg, n (%)				**0.46**
<120	30,561 (28.3)	162 (18.4)	30,399 (28.4)	
120–129	23,530 (21.8)	120 (13.7)	23,410 (21.8)	
130–139	27,681 (25.6)	175 (19.9)	27,506 (25.7)	
140–159	16,397 (15.2)	233 (26.5)	16,164 (15.1)	
160–179	4698 (4.3)	108 (12.3)	4590 (4.3)	
≥180	1461 (1.4)	47 (5.3)	1414 (1.3)	
Unknown	3763 (3.5)	34 (3.9)	3729 (3.5)	
**Baseline laboratory values**				
CKD-EPI eGFR, Mean (SD), mL/min/1.73m^2^	46.3 (10.4)	25.0 (14.5)	46.5 (10.2)	**2.11**
CKD-EPI eGFR category, mL/min/1.73m^2^, n (%)				**2.91**
45–59	68,305 (63.2)	134 (15.2)	68,171 (63.6)	
30–44	30,452 (28.2)	117 (13.3)	30,335 (28.3)	
25–29	4254 (3.9)	58 (6.6)	4196 (3.9)	
20–24	2533 (2.3)	115 (13.1)	2418 (2.3)	
15–19	1597 (1.5)	187 (21.3)	1410 (1.3)	
10–14	753 (0.7)	212 (24.1)	541 (0.5)	
5–9	182 (0.2)	53 (6.0)	129 (0.1)	
<5	15 (0.0)	3 (0.3)	12 (0.0)	
Hemoglobin, g/dL, n (%)				**0.26**
≥13	41,666 (38.5)	128 (14.6)	41,538 (38.7)	
12.0–12.9	20,103 (18.6)	130 (14.8)	19,973 (18.6)	
11.0–11.9	14,319 (13.2)	217 (24.7)	14,102 (13.2)	
10.0–10.9	6925 (6.4)	173 (19.7)	6752 (6.3)	
9.0–9.9	2677 (2.5)	81 (9.2)	2596 (2.4)	
<9.0	1288 (1.2)	51 (5.8)	1237 (1.2)	
Unknown	21,113 (19.5)	99 (11.3)	21,014 (19.6)	
Albumin-to-creatinine ratio, converted, mg/g				
Median (interquartile range)	19.7 (9.0–81.0)	315.0 (151.8–1588.3)	19.1 (9.0–81.0)	**0.11**
Albumin-to-creatinine ratio, mg/g				
Median (interquartile range)	21.6 (7.8–87.7)	228.3 (55.2–250.0)	21.4 (7.8–86.4)	0.07
Missing, n (%)	55,435 (51.3)	574 (65.3)	54,861 (51.2)	
Protein-to-creatinine ratio, g/g				
Median (interquartile range)	0.6 (0.2–1.7)	3.6 (1.4–7.0)	0.6 (0.2–1.6)	**0.16**
Missing, n (%)	81,633 (75.5)	220 (25.0)	81,413 (75.9)	
Urine dipstick protein excretion				**0.80**
Negative	45,132 (41.8)	56 (6.4)	45,076 (42.0)	
Trace	20,090 (18.6)	70 (8.0)	20,020 (18.7)	
1+	15,126 (14.0)	135 (15.4)	14,991 (14.0)	
2+	8893 (8.2)	251 (28.6)	8642 (8.1)	
3+	4268 (3.9)	339 (38.6)	3929 (3.7)	
Unknown	14,592 (13.5)	128 (14.6)	14,464 (13.5)	

Abbreviations: RRT, renal replacement therapy; SD, standard deviation; ACE, angiotensin converting enzyme; CKD-EPI, Chronic Kidney Disease Epidemiology Collaboration; eGFR, estimated glomerular filtration rate

*Cohen’s R was calculated for medians while Cohen’s D was calculated for means and categorical variables. For both Cohen’s R and Cohen’s D, we considered a value ≥ 0.10 to be indication of a significant difference between comparison groups.

### One-year risks of ESKD, receipt of hospice services and death

During 1-year follow-up among 108,091 patients in the KFRE cohort, 879 (0.81%, 95% CI:0.76–0.87%) progressed to ESKD, 1,133 (1.05%, 95% CI:0.99–1.11%) received hospice services before ESKD or death, and 4,635 (4.29%, 95%CI:4.23–4.35%) died before ESKD (Table 2). As expected, the risk of each outcome was higher with more severe baseline CKD, particularly for the outcome of ESKD (Table 2).

**Table 2:** Crude 1-year risks of ESKD, utilization of hospice services, and death among eligible adults with chronic kidney disease, January 2008—September 2016, overall and stratified by CKD stage.

Outcome	KFRE Analytic Cohort		KPNW Equation Analytic Cohort	
	N (Total Patients)	Risk, % (95% CI)	N (Total Patients)	Risk, % (95% CI)
**ESKD**				
All CKD	879 (108,091)	0.81 (0.76–0.87)	757 (84,449)	0.90 (0.83–0.96)
Stage 3	251 (98,757)	0.25 (0.22–0.29)	209 (76,476)	0.27 (0.24–0.31)
Stage 4	360 (8,384)	4.29 (3.86–4.73)	313 (7,125)	4.39 (3.92–4.87)
Stage 5	268 (950)	28.2 (25.4–31.1)	235 (848)	27.7 (24.7–30.7)
**Hospice utilization before ESKD**				
All CKD	1,133 (108,091)	1.05 (0.99–1.11)	1,042 (84,449)	1.23 (1.16–1.31)
Stage 3	1,010 (98,757)	1.02 (0.96–1.09)	931 (76,476)	1.22 (1.14–1.30)
Stage 4	104 (8,384)	1.23 (0.99–1.46)	92 (7,125)	1.29 (1.03–1.55)
Stage 5	19 (950)	2.00 (1.11–2.89)	19 (848)	2.24 (1.24–3.24)
**Death before ESKD**				
All CKD	4,635 (108,091)	4.29 (4.23–4.35)	4,055 (84,449)	4.80 (4.66–4.95)
Stage 3	4,076 (98,757)	4.13 (4.00–2.25)	3,568 (76,476)	4.67 (4.52–4.81)
Stage 4	489 (8,384)	5.83 (5.33–6.33)	424 (7,125)	5.95 (5.40–6.50)
Stage 5	70 (950)	7.37 (5.71–9.03)	63 (848)	7.43 (5.66–9.19)

The KPNW equation cohort displayed similar patterns across CKD stages in all outcomes, with slightly higher absolute risks than in the KFRE cohort (Table 2). During 1-year follow-up among 84,449 patients in the KPNW equation cohort, 757 (0.90%, 95% CI:0.83–0.96%) progressed to ESKD, 1,042 (1.23%, 95% CI:1.16–1.31%) received hospice services before ESKD or death, and 4,055 (4.80%, 95% CI:4.66–4.95%) died before ESKD (Table 2).

### CKD stage-specific utility for predicting 1-year ESKD risk

**Fig 2:**
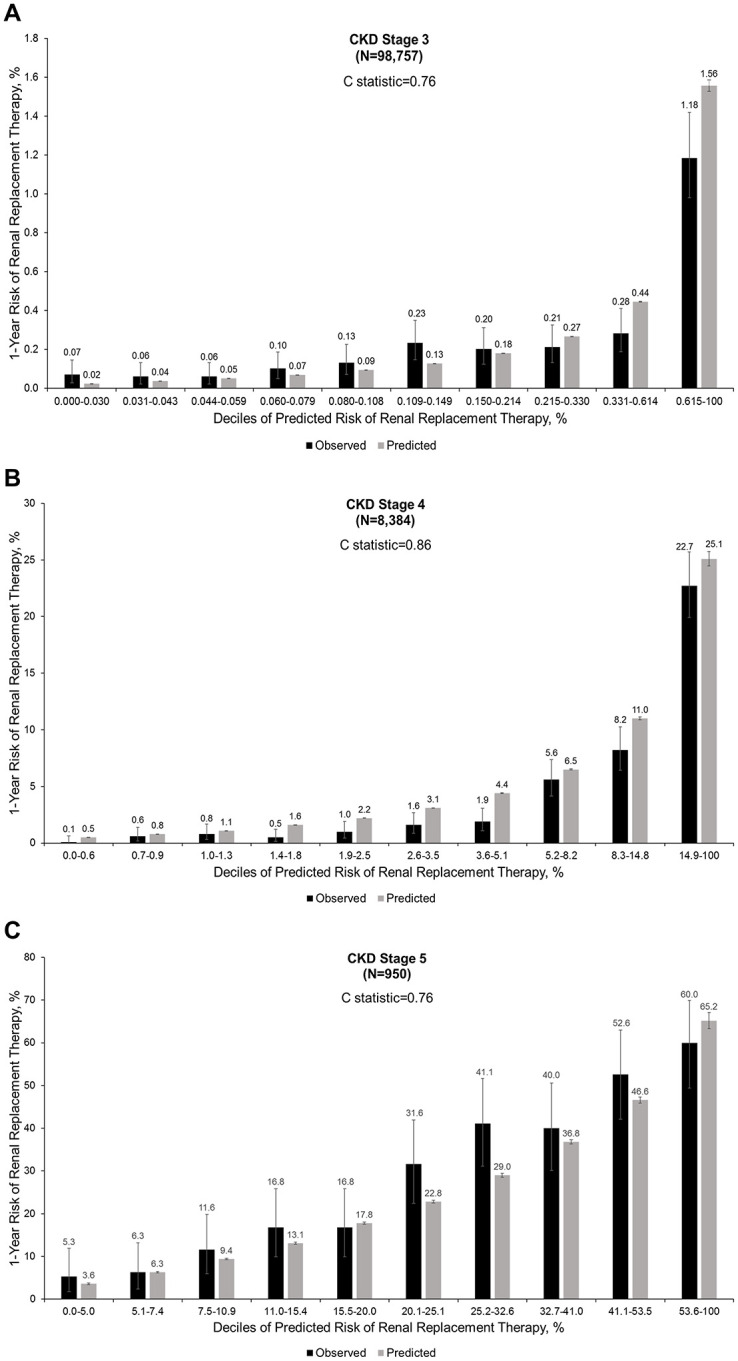
Calibration plot of observed versus predicted 1-year risk of initiating chronic renal replacement therapy by CKD stage for the KFRE.

KFRE displayed only moderate discrimination for CKD Stage 3 and Stage 5 patients (c = 0.76 for both groups), with much better discrimination for CKD Stage 4 patients (c = 0.86) (Fig 2). Overall equation calibration of the KFRE was relatively good for CKD Stages 3 and 4, with modest overestimation of risk, but KFRE consistently underestimated the actual 1-year risk of ESKD in CKD Stage 5 patients. Furthermore, in CKD Stage 3 and 4 patients, the overestimation of actual risk increased as the predicted risk increased, while in CKD Stage 5 patients, the highest underestimation was in patients with predicted risk between 20% and 32% (Fig 2).

**Fig 3:**
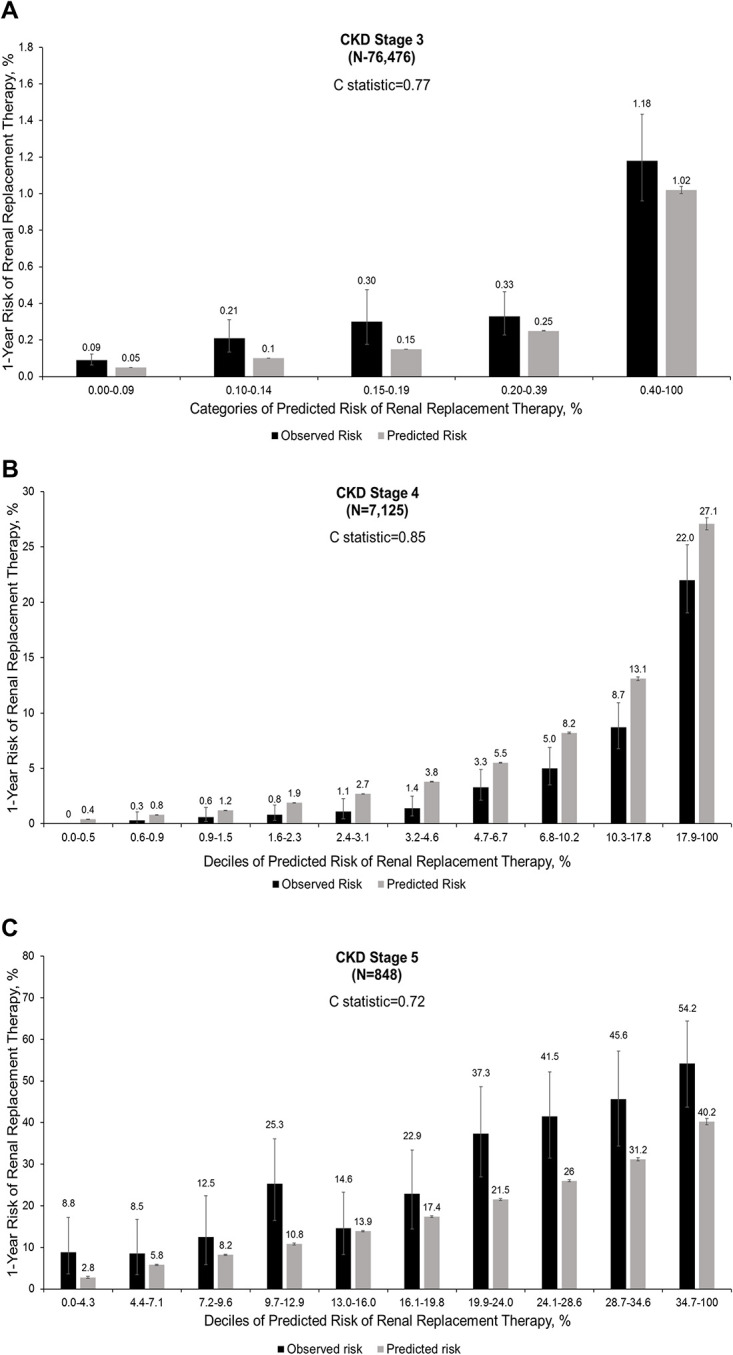
Calibration plot of observed versus predicted 1-year risk of initiating chronic renal replacement therapy by CKD stage for the KPNW equation.

The KPNW equation displayed very similar discrimination as the KFRE in CKD Stages 3 and 4 patients (c = 0.77 and 0.85, respectively), with poorer discrimination for CKD Stage 5 (c = 0.72). There was slight underestimation of actual 1-year ESKD risk in CKD Stage 3 patients but greater overestimation of ESKD risk in CKD Stage 4 patients. Calibration was notably worse in CKD Stage 5 patients, with significant underestimation of risk of up to 15.8% among patients with a predicted risk between 20% and 24% (Fig 3).

We also calculated Brier scores for each equation in each CKD stage to assess calibration (Table 3). Within each CKD stage, KFRE had better calibration than the KPNW equation.

**Table 3:** Brier scores describing calibration for each ESKD prediction equation by CKD stage.

CKD Stage	KFRE Cohort		KPNW Equation Cohort	
	Risk of ESKD (%)	Brier Score	Risk of ESKD (%)	Brier Score
Stage 3	0.25%	0.0025	0.27%	0.0027
Stage 4	4.29%	0.0359	4.39%	0.0381
Stage 5	28.21%	0.1708	27.71%	0.1864

## Discussion

Within a large, contemporary, ethnically diverse population with CKD Stages 3–5, we found notably higher 1-year risks of ESKD with higher CKD stage, as expected, with a very low overall 1-year risk of ESKD in all CKD Stage 3 patients (0.25–0.27%) compared with more than one in four CKD Stage 5 patients within 1 year (27.7–28.2%). Across these ranges of absolute risk, it is important to identify more accurately the subset of patients who are at high risk for initiating RRT within the next year. Importantly, the KFRE and KPNW equation showed only moderate ability to discriminate between relatively higher vs. lower risk patients, particularly for CKD Stage 3 (c = 0.76–0.77) and Stage 5 (c = 0.72–0.76), and these estimates are notably lower than those previously reported among combined CKD 3–5 populations (c range: 0.84–0.91) [7, 8, 26, 31].

With regards to predicting absolute 1-year ESKD risk in CKD Stage 3 patients, equation calibration was of variable accuracy which reflects the challenge of predicting a rare event in this low-risk population, especially given the significantly higher crude risk of all-cause death and the higher rate of hospice care before any RRT. Importantly, the KFRE underestimated actual 1-year risk of ESKD among the lower deciles of predicted risk but overestimated ESKD risk among the higher deciles of predicted risk. In contrast, the KPNW equation underestimated the 1-year risk of ESKD across the entire spectrum of predicted risk.

Among CKD Stage 4 patients, the overall 1-year risk of ESKD was 4.3%, which was higher than the probability of entering hospice care but lower than the crude risk of dying before initiating RRT. Both KFRE and the KPNW equation had significantly better discrimination (c = 0.86 and 0.85, respectively), with good calibration across the spectrum of predicted risk and only modest overestimation of actual 1-year ESKD risk. Notably, the additional variables used in the KPNW equation (i.e., systolic blood pressure, anti-hypertensive medication usage, hemoglobin level, and presence of diabetes with complications) did not appear to provide incremental improvement in discrimination or calibration for 1-year ESKD above the demographic characteristics and kidney measures (eGFR, uACR) that were common between KPNW and KFRE equations.

Not surprisingly, CKD Stage 5 patients had the highest crude risk of initiating chronic RRT within 1 year (28.2%), which was much higher than the crude risks of either receiving hospice care or dying before initiating RRT. These data also highlight that a large fraction of CKD Stage 5 patients remain alive and not receiving RRT or hospice care within one year, supporting the need to better risk stratify these patients with eGFR <15 ml/min/1.73 m^2^ in informing decisions around issues like timing of vascular access or peritoneal dialysis catheter placement. Focusing resources on the subset of CKD Stage 5 patients most likely to need RRT in the short-term could also be a more cost-effective population strategy. Given that both the KFRE and KPNW equation had only modest discrimination (0.76 and 0.72, respectively) and significant underestimation of the actual risk of initiating RRT, even more accurate prediction equations are needed.

Current KDIGO guidelines [10] recommend timely referral for planning RRT in people with progressive CKD in whom the risk of kidney failure within 1 year is 10–20% or higher, as determined by validated risk prediction tools. Currently, there are no validated 1-year ESKD prediction models [32] to assist physicians and patients in facilitating timelier referral for transplant evaluation, earlier identification of potential living donors, promoting early education of home dialysis options, and more appropriate, timely referral for permanent vascular access or peritoneal catheter placement. Identifying the subset of patients in each CKD stage at highest short-term risk of developing ESKD would also help with targeting optimization of the use and dosing of angiotensin-converting enzyme inhibitors/angiotensin II receptor blocker therapy, and sodium-glucose cotransporter-2 (SGLT-2) Inhibitors and non-steroidal mineralocorticoid receptor antagonists, along with avoidance of nephrotoxin exposures as well as reducing placement of futile vascular access. Accurate short-term ESKD risk information would also promote timely, relevant discussions with high-risk patients about their preferences for different RRT options vs. conservative management approaches, including decisions about end-of-life care.

Given the observed limitations of KFRE and KPNW equations, we propose several approaches to improve the utility of risk prediction equations for current clinical practice. First, CKD stage-specific prediction models should be developed and validated given that there are substantial differences in the comorbidity burden and duration of time spent in different stages of CKD severity. This is highlighted by the variability in performance of the KFRE and KPNW equations across CKD stages, with their best performance only in CKD Stage 4 patients. Second, models should account for patients with missing values of covariates to facilitate clinical management in parallel with efforts to obtain the missing data. In our cohort, 31,919 patients (22.7%) were excluded from the KFRE analytic cohort because of missing proteinuria measurements, and an additional 25,338 (18.1%) were excluded from the KPNW equation analytic cohort because of missing hemoglobin or systolic blood pressure measurements (Fig 1). These represent a large proportion of all eligible CKD patients, some of whom may be at high risk for progression to ESKD and would otherwise not be identified even with partially available information on other variables. Lastly, prediction models should consider other potentially important risk factors for progression to ESKD beyond the limited candidate variables examined in the development of KFRE and KPNW equations. For example, in our cohort, differences were noted for characteristics such as prior AKI and chronic liver disease between patients who did or did not initiate RRT within one year (Table 1). However, the 8-variable KPNW equation did not have improved predictive ability compared to the 4-variable KFRE, suggesting that not all risk factors will result in meaningful performance improvements across diverse populations. With the increasingly widespread use of EHR systems nationally, ascertaining data on complex comorbidity and other patient characteristics is becoming more feasible on a large scale and in real time to facilitate more accurate, timely short-term ESKD risk prediction.

Finally, we observed that patients in CKD 3 and CKD 4 had a higher 1-year risk of death than ESKD, which is consistent with another study showing death is more common than reaching dialysis in CKD stages 2, 3, and 4 patients over a 5-year observation period [33]. In contrast, among CKD 5 patients, 1-year risk of ESKD was fourfold higher than death. This suggests that clinical management and patient education efforts should focus differently in CKD 3 and 4 vs. CKD 5. For example, the priority should likely be on reducing cardiovascular risk over ESKD risk for CKD stage 3 and 4 patients but more on preparing for KRT in CKD 5 patients in parallel with cardiovascular risk reduction.

Our study has several strengths. We studied a large, socio-demographically diverse population of eligible patients across the spectrum of CKD severity which allowed us to examine the utility (as reflected by estimates of discrimination and calibration) of both models within each CKD stage in the same analysis population. Unlike previous studies, we applied stricter eGFR criteria by requiring multiple outpatient, non-emergency department eGFR measurements <60 mL/min/1.73m^2^ to ensure patients had CKD. Given our comprehensive EHR system, we were also able to capture information on relevant comorbidities and laboratory measurements to characterize CKD patients who did or did not progress to ESKD within a short time frame.

Our study also had several limitations. Our method of calculating 1-year risks from the KPNW equation involved assuming a constant hazard for 2-year risks and dividing that estimate in half. Given that non-linear trajectories in kidney function can occur in certain CKD patients [34], our assumption may contribute to a less accurate estimate. However, we believe that this approach reflects how the KPNW equation would be used in clinical practice if a physician was trying to estimate a patient’s ESKD risk at 1 year. This also supports the need to develop and validate separate and more accurate equations across a range of follow-up periods to allow for more effective individual-level and population-level management strategies. This is particularly relevant given the expansion nationally in the use of CKD population management programs [35], along with the recent focus on more optimal KRT starts [36]. Imputation of uACR values may cause equations to be less accurate than originally intended, although the presence of missing data may reflect performance in real world EHR settings. Finally, we conducted our study among CKD patients receiving care within an integrated healthcare delivery system in Northern California, so our results may not be fully generalizable to uninsured patients or to other types of practice settings or geographic areas.

In conclusion, we found that existing ESKD risk prediction equations have variable utility by stage of CKD for predicting 1-year risk of initiating RRT within a diverse community-based population. Additional efforts are needed to provide even more accurate, personalized, stage-specific estimates of the risk of ESKD across short- and long-term time periods to facilitate more optimal CKD management and planning for individuals and populations.

## Supporting information

S1 Table Baseline characteristics of 84,449 adults with chronic kidney disease eligible for the KPNW equation, January 1, 2008-September 30, 2015, overall and stratified by initiation of renal replacement therapy (RRT) within 1 year of follow-up. (DOCX)
