# Supplementary material for: CKD stage-specific utility of two equations for predicting 1-year risk of ESKD
Source: PLoS One. 2023 Nov 1;18(11):e0293293. doi: 10.1371/journal.pone.0293293 (PMC10619781; doi:10.1371/journal.pone.0293293)
Supplement: S1 Table — (DOCX) [file pone.0293293.s001.docx]

**Supplementary Table 1. Baseline characteristics of 84,449 adults with chronic kidney disease eligible for the KPNW equation, January 1, 2008-September 30, 2015, overall and stratified by initiation of renal replacement therapy (RRT) within 1 year of follow-up.**

| **Characteristic** | **Overall** | **ESKD at 1 year** | **No ESKD at 1 year** | **Standardized difference*** |
| --- | --- | --- | --- | --- |
|  | **(N=84,449)** | **(N=757)** | **(N=83,692)** |  |
| Age, Mean (SD), years | 75.0 (11.0) | 62.5 (14.4) | 75.1 (10.9) | **0.99** |
| Age Category, years, n (%) |  |  |  | **0.12** |
| 18-40 | 581 (0.7) | 54 (7.1) | 527 (0.6) |  |
| 41-60 | 7276 (8.6) | 254 (33.6) | 7022 (8.4) |  |
| 61-75 | 30,301 (35.9) | 294 (38.8) | 30,007 (35.9) |  |
| >75 | 46,291 (54.8) | 155 (20.5) | 46,136 (55.1) |  |
| Women | 46,853 (55.5) | 355 (46.9) | 46,498 (55.6) | **0.17** |
| Self-reported Race, n (%) |  |  |  | **0.44** |
| White | 53,665 (63.5) | 287 (37.9) | 53,378 (63.8) |  |
| Black | 6396 (7.6) | 115 (15.2) | 6281 (7.5) |  |
| Asian/Pacific Islander | 9859 (11.7) | 155 (20.5) | 9704 (11.6) |  |
| Other | 14,312 (16.9) | 199 (26.3) | 14,113 (16.9) |  |
| Unknown | 217 (0.3) | 1 (0.1) | 216 (0.3) |  |
| Hispanic ethnicity, n (%) | 10,441 (12.4) | 180 (23.8) | 10,261 (12.3) | **0.3** |
| Smoking status, n (%) |  |  |  | **0.11** |
| Current smoker | 4368 (5.2) | 79 (10.4) | 4289 (5.1) |  |
| Former smoker | 33,331 (39.5) | 262 (34.6) | 33,069 (39.5) |  |
| Nonsmoker | 46,750 (55.4) | 416 (55.0) | 46,334 (55.4) |  |
| **Medical history, n (%)** |  |  |  |  |
| Prior acute kidney injury | 14,155 (16.8) | 255 (33.7) | 13,900 (16.6) | **0.40** |
| Acute myocardial infarction | 3433 (4.1) | 47 (6.2) | 3386 (4.0) | **0.10** |
| Heart failure | 13,543 (16.0) | 159 (21.0) | 13,384 (16.0) | **0.13** |
| Ischemic stroke or transient ischemic attack | 4023 (4.8) | 41 (5.4) | 3982 (4.8) | 0.03 |
| Peripheral artery disease | 7403 (8.8) | 51 (6.7) | 7352 (8.8) | 0.08 |
| Mitral and/or aortic valvular disease | 8022 (9.5) | 70 (9.2) | 7952 (9.5) | 0.01 |
| Atrial fibrillation or flutter | 12,876 (15.2) | 74 (9.8) | 12,802 (15.3) | **0.17** |
| Venous thromboembolism | 1260 (1.5) | 9 (1.2) | 1251 (1.5) | 0.03 |
| Other thromboembolic events | 1080 (1.3) | 18 (2.4) | 1062 (1.3) | 0.08 |
| Coronary artery bypass surgery | 1957 (2.3) | 21 (2.8) | 1936 (2.3) | 0.03 |
| Percutaneous coronary intervention | 3449 (4.1) | 45 (5.9) | 3404 (4.1) | 0.08 |
| Diabetes mellitus | 39,128 (46.3) | 500 (66.1) | 38,628 (46.2) | **0.41** |
| Diabetes Complications Severity Index |  |  |  | **0.12** |
| No Diabetes | 45,321 | 257 | 45064 |  |
| 0 complication | 14,482 (17.1) | 117 (15.5) | 14,365 (17.2) |  |
| 1 complication | 13,166 (15.6) | 177 (23.4) | 12,989 (15.5) |  |
| 2 complications | 7570 (9.0) | 131 (17.3) | 7439 (8.9) |  |
| > 3 complications | 3910 (4.6) | 75 (9.9) | 3835 (4.6) |  |
| Hypertension | 73,830 (87.4) | 682 (90.1) | 73,148 (87.4) | 0.09 |
| Dyslipidemia | 69,990 (82.9) | 639 (84.4) | 69,351 (82.9) | 0.04 |
| Hyperthyroidism | 4651 (5.5) | 30 (4.0) | 4621 (5.5) | 0.07 |
| Hypothyroidism | 18,314 (21.7) | 118 (15.6) | 18,196 (21.7) | **0.16** |
| Chronic liver disease | 2564 (3.0) | 71 (9.4) | 2493 (3.0) | **0.27** |
| Chronic lung disease | 22,090 (26.2) | 162 (21.4) | 21,928 (26.2) | **0.11** |
| Diagnosed dementia | 4589 (5.4) | 7 (0.9) | 4582 (5.5) | **0.26** |
| Diagnosed depression | 15,093 (17.9) | 117 (15.5) | 14,976 (17.9) | 0.06 |
| Hospitalized bleed | 3647 (4.3) | 39 (5.2) | 3608 (4.3) | 0.04 |
| Body mass index, Mean (SD), kg/m^2^ | 28.9 (6.5) | 30.0 (7.6) | 28.9 (6.5) | **0.16** |
| Body mass index category, kg/m^2^, n (%) |  |  |  | 0.07 |
| <18.5 | 1272 (1.5) | 5 (0.7) | 1267 (1.5) |  |
| 18.5-24.9 | 22,424 (26.6) | 205 (27.1) | 22,219 (26.5) |  |
| 25-29.9 | 29,079 (34.4) | 224 (29.6) | 28,855 (34.5) |  |
| 30-39.9 | 25,714 (30.4) | 251 (33.2) | 25,463 (30.4) |  |
| ≥40 | 3549 (4.2) | 56 (7.4) | 3493 (4.2) |  |
| Unknown | 2411 (2.9) | 16 (2.1) | 2395 (2.9) |  |
| Systolic blood pressure, Mean (SD), mmHg | 129.6 (18.0) | 139.5 (23.2) | 129.5 (17.9) | **0.48** |
| Systolic blood pressure category, mmHg, n (%) |  |  |  | **0.37** |
| <120 | 25,183 (29.8) | 153 (20.2) | 25,030 (29.9) |  |
| 120-129 | 18,719 (22.2) | 108 (14.3) | 18,611 (22.2) |  |
| 130-139 | 21,982 (26.0) | 155 (20.5) | 21,827 (26.1) |  |
| 140-159 | 13,520 (16.0) | 210 (27.7) | 13,310 (15.9) |  |
| 160-179 | 3865 (4.6) | 91 (12.0) | 3774 (4.5) |  |
| ≥180 | 1180 (1.4) | 40 (5.3) | 1140 (1.4) |  |
| Unknown |  |  |  |  |
| **Baseline laboratory values** |  |  |  |  |
| CKD-EPI eGFR, Mean (SD), mL/min/1.73m^2^ | 46.0 (10.6) | 24.6 (14.5) | 46.2 (10.4) | **1.71** |
| CKD-EPI eGFR category, mL/min/1.73m^2^, n (%) |  |  |  | **1.79** |
| 45-59 | 52,534 (62.2) | 114 (15.1) | 52,420 (62.6) |  |
| 30-44 | 23,942 (28.4) | 95 (12.5) | 23,847 (28.5) |  |
| 25-29 | 3544 (4.2) | 50 (6.6) | 3494 (4.2) |  |
| 20-24 | 2169 (2.6) | 92 (12.2) | 2077 (2.5) |  |
| 15-19 | 1412 (1.7) | 171 (22.6) | 1241 (1.5) |  |
| 14-Oct | 674 (0.8) | 186 (24.6) | 488 (0.6) |  |
| 9-May | 160 (0.2) | 47 (6.2) | 113 (0.1) |  |
| <5 | 14 (0.0) | 2 (0.3) | 12 (0.0) |  |
| Hemoglobin, g/dL, n (%) |  |  |  | **0.24** |
| ≥13 | 40,217 (47.6) | 122 (16.1) | 40,095 (47.9) |  |
| 12.0-12.9 | 19,587 (23.2) | 127 (16.8) | 19,460 (23.3) |  |
| 11.0-11.9 | 13,967 (16.5) | 215 (28.4) | 13,752 (16.4) |  |
| 10.0-10.9 | 6805 (8.1) | 167 (22.1) | 6638 (7.9) |  |
| 9.0-9.9 | 2615 (3.1) | 78 (10.3) | 2537 (3.0) |  |
| <9.0 | 1258 (1.5) | 48 (6.3) | 1210 (1.4) |  |
| Unknown |  |  |  |  |
| Albumin-to-creatinine ratio, converted, mg/g |  |  |  |  |
| Median (interquartile range) | 20.8 (9.0-81.0) | 335.9 (139.4-1661.0) | 20.1 (9.0-81.0) | **0.80** |
| Albumin-to-creatinine ratio, mg/g |  |  |  |  |
| Median (interquartile range) | 22.3 (8.0-89.0) | 186.0 (48.1-250.0) | 22.1 (8.0-87.7) | **0.84** |
| Missing, n (%) | 45,694 (54.1) | 514 (67.9) | 45,180 (54.0) |  |
| Protein-to-creatinine ratio, g/g |  |  |  |  |
| Median (interquartile range) | 0.6 (0.2-1.6) | 3.4 (1.3-6.7) | 0.6 (0.2-1.5) | **0.13** |
| Missing, n (%) | 63,419 (75.1) | 192 (25.4) | 63,227 (75.5) |  |
| Urine dipstick protein excretion |  |  |  | **0.18** |
| Negative | 36,397 (43.1) | 51 (6.7) | 36,346 (43.4) |  |
| Trace | 16,662 (19.7) | 66 (8.7) | 16,596 (19.8) |  |
| 1+ | 12,146 (14.4) | 122 (16.1) | 12,024 (14.4) |  |
| 2+ | 6967 (8.2) | 225 (29.7) | 6742 (8.1) |  |
| 3+ | 3229 (3.8) | 273 (36.1) | 2956 (3.5) |  |
| Unknown | 9048 (10.7) | 20 (2.6) | 9028 (10.8) |  |
